# A General Outline of the Treatment of Eczema

**Published:** 1890-07

**Authors:** George T. Elliot

**Affiliations:** Dermatologist to Demilt Dispensary and the New York Infant Asylum; Assistant Physician New York Skin and Cancer Hospital, etc.


### A GENERAL OUTLINE OF THE TREATMENT OF ECZEMA.

---

BY GEORGE T. ELLIOT, M. D.,

Dermatologist to Demilt Dispensary and the New York Infant Asylum; Assist-ant Physician New York Skin and Cancer Hospital, etc.

---

*(Concluded from last issue.)*

In the large majority of cases, however, local treatment will be also required. Moreover, this latter is all that will be necessary for a cure in a very large number of instances—the trade eczemas for example—and there are some forms of the disease absolutely uninfluenced by internal medication—those parasitic in origin. In consequence, the local remedies useful in eczema are of the greatest importance, but as their number is legion, I shall confine myself to those only which have proved especially beneficial in my hands. The choice of local applications will depend upon whether an eczema is acute or chronic and will have also to be varied according to the for mo[illegible] the disease presented by the patient. In acute eczema the object to be attained from the external treatment is protection from irritation of all kinds, subsidence of the inflammation and absorption of the exudation. For these purposes either powders, lotions or ointments may be used. Should the eruption be vesicular or erythematous in character and accompanied by much œdema, swelling and heat, the formation of crusts not yet having occurred, finely powdered starch, corn starch, flour or any similar indifferent substance may be used. For obvious reasons these are not applicable on the face and hairy scalp, but are more suitable for other portions of the body. The powder should be applied very thickly over the affected surface and be frequently renewed. If the eruption is on an extremity, a stocking or linen bag filled with the starch may be drawn over it, but if the trunk or the entire body is implicated, it will be better to put the patient bed and strew the powder over him so that he is covered with to the depth of ¼ inch or more. Care should be taken not ıly to have contiguous surfaces, axilla, etc., well separated by e powder, but also by the interposition of pledgets of cotton. ıstead of the powders, lotions and ointments may be employed, d they are especially indicated when there is much weeping or usting. If the former condition exists, lotions are to be pre- rred and subsequently an ointment, but if there is much crust- g, ointments from the very start will be the best. The lotions hich can be recommended are the dilute lead wash or a freshly ıade lactate of lead (liq. Pb. subacetat. dil. ℨi, fresh ıilk ℥i), especially if the eczema is erythematous in character. or children, an emulsion of almonds, to which a few grains of od. biborat. (gr. iii to v ad. ℥i) has been added, will be very oothing, but it must be made fresh every day, as it quickly be- omes rancid and will then irritate. Any other emollient may e used, but usually the benefit derived from them is only tem- orary, and applications which are somewhat astringent are more ecessary. Lotions which possess such property contain zinc, ismuth, alum, etc., and a very useful one will be the lotio alamin. et zinc oxid:

℞. Pulv. Calaminaris.
Pulv. zinc oxide.......................*aa* gr. xv.
Aq. aurant. flor...............................℥i.

To this glycerine may be added (ℨss ad. ℥i), if the effect of ıe lotion is to increase the itching, and it may also serve as an xcipient for certain other substances, should such be indicated r desired—ichthyol, resorcin, etc. When the eczematous sur- ace is very moist, I have usually substituted carbonate of mag- esia for Calamine, owing to the former's greater hygroscopic ower. From grs. i to grs. xx to the ℥i will be sufficient. In uch cases where the inflammation is less acute in character, the fficacy of the lotion will be greatly increased by the addition of chthyol (gr. x or xv to ℥i). This lotion of zinc oxide and nagnesia will also prove particularly good in that severe and roublesome eczema caused by rhus toxicodendron, especially if esorcin gr. x ad. ℥i be added to it.

On the scalp, oily applications can be used, such as salicylated oil (salicylic acid grs. v ad. ʒi) or carbolic oil, but their value in acute eczema is a limited one, they being more especially indicated when thick crusting is to be removed prior to the application of an ointment or lotion. When washes are used, they must be freely applied and allowed to dry on the affected surfaces, they should be reapplied three or four times daily, or oftener if necessary. If there is much weeping, it will be better to cover the diseased portion of the skin with a piece of canton flannel, or sheet lint, or a layer of absorbent cotton soaked in the lotion and kept moist by pouring on some of the fluid every half hour or so. Care must be taken, both in using lotions and also ointments, to thoroughly separate contiguous surfaces from each other. If that is not done, there is retention of the exudation, friction and maceration of the skin occurs, and the eczema is kept up indefinitely. Lotions not infrequently produce a certain degree of irritation after a few days' use or even from the very first. When such is the case, an ointment should be substituted and, in fact, salves are generally the most grateful and beneficial of all applications for eczema. Many things have been proposed and many attempts have been made to dispense with them, but they have nevertheless, asserted and retained their position. In cases of acute eczema, only the mildest and most non-irritating ointments should be used, such as the officinal oxide of zinc, or fresh lard alone, or mixed with an equal part of starch. When nothing else is at hand, boiled starch applied cold, in the form of a poultice will do well, and in very moist eczemas has given me excellent results. It should be changed every four or five hours. Complaint is often made that it feels too drying and produces discomfort after a time. If that should be the case, the application of starch may be alternated with that of lard or any fresh grease. The benefit obtained from any one of these unguenta will be greater, however, if carbolic acid (grs. v--℥i) or boric acid (grs.30–60 ℥i) or salicylic acid be added. The most generally applicable ointment in acute eczema, and one which is suitable for all cases is:

℞. Acid. salicyl...........................gr. x-xx.
Pulv. zinc. oxid.
Amyli.................................*aa* ℨiv.
Ungt. zinc. oxid.........................℥i.
*M.* et S.

Should the exudative symptoms be not very marked, the amylum and oxide of zinc may be omitted, but when there is much moisture over the affected surface, the efficacy of the ointment can be greatly increased by substituting for the starch twenty to thirty grains or more of carbonate of magnesia. Another ointment at times useful is one consisting of

Pulv. Calaminaris.
Pulv. zinc oxydat.....................*aa* grs. 20.
Adipis.................................℥i.
*M.* et S.

and to it may be added any other ingredients, as salicylic acid or carbolic acid, etc. One of the most useful of these salves has been found by me to be one consisting of acid salicyl. gr. x, acid boric grs. 30, ungt.zinc oxide ℥. In the use of these ointments care must be taken to see that they are made of fresh materials and that they are sweet when applied. An old and rancid ointment acts as an irritant to the already inflamed skin, owing to the presence of the fatty acids and will only increase the intensity and extent of the disease; vaseline also is not to be recommended as a base, as it prevents almost entirely the action of any drug incorporated with it, often irritates and is of little value when absorption is desired. Lard is by all means the best excipient and should be ordered alone or with wax, as in the ungt. simp., aq. rosæ, etc. In acute eczema, the ointment should be applied at least twice in twenty-four hours. If the crusting on the affected surface is marked, it ought first to be removed by means of the salicylated or carbolized oil, but if only slight, the salve will be sufficient for the purpose.

The manner in which ointments are used will, however, influence their value and efficacy to a great degree in all of the forms and stages of eczema. On hairy surfaces, of course, they have to be rubbed on, their retention being facilitated by the hair, but on all other portions of the body a similar method of application is of questionable benefit, the ointment being in a very short space of time absorbed by the clothing, or rubbed off upon everything with which the patient comes in contact; the affected surface is left unprotected and exposed anew to all sorts of irritation. To prevent such occurrences and to keep up a constant action upon the diseased skin, the unguent should, therefore, b[illegible] spread out from ⅛ to ¼ of an inch thick upon pieces of lint o[illegible] canton flannel, cut so as to fit the affected surface, and be retained in place by means of a bandage. In the case of the face a mask, with necessary apertures for the eyes and mouth, can be made. If it is the hands that are to be treated, then every finger should be done up separately. When the ointments are to be applied freshly there is no need to remove the portion which remains adherent to the affected surface, by washing or other means. A portion may be rubbed off with a little cotton, but only if it has caked thickly on the surface should it be taken off. Under these circumstances, the use of a little oil quickly loosens and softens the encrusted mass. Instead of using the lint or flannel, the ointment may also be spread thickly over the affected surface, and then covered with a layer of absorbent cotton kept in place by a gauze or other bandage.

Should the acute outbreak of eczema be a pustular one, the treatment is somewhat different, owing to the fact that, together with the catarrhal inflammation, there is also a local infection with the micro-organisms which produce pus. In consequence, antiseptics are called for, but not such as the mercuric chloride or iodoform, as they have themselves the property of causing eczema, but milder ones are indicated. A 5 to 10 per cent. salicylic acid ointment, or a 2 to 4 per cent. carbolic one, or boric acid (ʒij ad. ℥i) can be used, but the best results obtained by me have been from ichthyol. I have either added it to one of the ointments already given or used it in the form of an aqueous or oily lotion. Should there be with the pustular outbreak much exudation, the following ointment will prove useful :

℞. Acid Salicyl.......................gr. x.
Ichthyol.........................grs. v–xxv.
Magnesiæ Carb....................grs. 25.
Ungt. oxid. zinci...................℥i.
*M.* et S.

Ichthyol may be added to the calamine et oxide of zinc lotion, to simple water in the strength of 1 to 5 or more per. cent. the scalp is the portion affected, it will be very useful in the rm of an oil—ichthyol grs. x–xxv ol. oliv. or lini ℥i. Espe-ally good and grateful to the patient is the application, when ne following formula is used:

℞. Ichthyol...........................gr. x–xxv.
Aq. Calcis.
Ol. amydal dulc....................*aa* ℨiv.
*M.* et S.

s it is exceedingly cooling and pleasant. When a pustular czema is present on the scalp, there are very commonly pedi-uli capitis, and in such cases the parasites must primarily be estroyed. This can best be done by means of equal parts of erosene and linseed oil, as it possesses the advantage over all ther remedies of destroying the nits as well as the lice. The onstant application of the kerosene for twenty-four hours will sually be sufficient and then the ichthyol oil or ointment can be sed. A more or less favorite remedy for eczema is sulphur. n acute cases of the disease it is absolutely contraindicated, and n fact is productive of more harm than good in all forms and tages of the disease, except in the seborrheic eczema, in which is certainly invaluable.

The object of the treatment of acute eczema as given has een that of reducing the active inflammation, of removing the xudation and of converting the vesicular, pustular or other ariety of the disease, into a simple squamous one. By means f the ointments and other applications, it may happen that the czema will not only subside, but even disappear altogether, hough more often the affected surface will remain itchy, irrita-le, and scaly, with a tendency to relapse and to pass gradually nto the chronic stage, with more or less thickening of the skin.

Under such circumstances, a change in the remedies will be imperatively necessary. Should the itching be the most prominent symptom, a mild tar ointment will be best. ℞. Pix liquida gr. xx–xl. ungt. zinc. oxid. ℥i. If there is still a little weeping and a deficient formation of good horny epidermis, then a 1 to 2 per cent. ichthyol ointment will be advisable, but when there is simply a slight irritability and roughness of the skin, a 1 per cent. carbolic, or a 3 per cent. boric acid, or a 2 per cent. tannic acid ointment, or the ungt. diachyli of Hebra will be sufficient. In using these latter, simply anointing the skin well will be all that is necessary. Their application should be kept up until the skin has regained it natural color, thickness and pliability, and at the same time protection from all irritation and the avoidance of water should be enjoined.

Turning now to the local treatment of the chronic forms of eczema, which constitute the bulk of the cases of the disease which come under observation, indications very different from those mentioned in acute outbreaks of the disease are encountered and dealt with. There may be a more or less great amount of weeping present, or the formation of vesicles, papules or pustules may go on in the same manner as when an acute outbreak exists, but there will be in addition a thickening and infiltration of the skin, not a simple inflammatory œdema, and also a most distressing itching. The importance of the thickened condition of the cutis is enormous and should under no circumstances be underestimated, for as long as any trace of it remains there is the liability of an outbreak supervening, and therefore no case of chronic eczema can be regarded as cured until this infiltration has been entirely removed. Relief from the distressing itching is also of necessity, not alone on account of comfort and ease to the patient, but because the scratching, which is induced by it, is one one of the most fruitful sources of the repeated acute relapses and exacerbations of a chronic eczema.

The local treatment must in consequence be directed against these two factors especially, and their removal must be made its object. To effect this, the employment of local means is beyond question important, for notwithstanding that any number of internal remedies have been suggested and recommended for the relief of the pruritus—gelsemium, cannabis indica, rumex, etc.,—yet in practice it has never been my good fortune to see any benefit result from their use, except when through the administration of some one of them, the patient is kept in a semi-narcotized condition, or is put to sleep. The local applications useful in chronic eczema consist especially of ointments. Only occasionally, and then under special conditions, are lotions or liniments of any value, while powders are practically useless.

The variety or form of chronic eczema most commonly met with, is the squamous. At the time the patient comes for consultation, it may be that the surface is simply scaly, without any symptoms of active inflammation, or there may be an irritable condition of the skin and the presence of a few or many points of weeping; or on the other hand, an entire denudation of the cutis of its horny epidermis and excessive serous exudation may be present; or perhaps an acute attack has been provoked upon the chronic and infiltrated surface. Each one of these conditions will therefore require special treatment, the primary object being to convert all of them into the simple squamous stage of the disease; in other words, to cause subsidence of the acute exacerbation, to promote formation of horny epidermis on the denuded and weeping points or patches, and to bring the eczema to a point where stimulating and other remedies can be applied, which will have the effect of removing the infiltration and of hastening its absorption. Should the symptoms, therefore, indicate an acute attack, any one of the applications recommended for acute eczema, in general, will be applicable. Should there be only some irritability and a little weeping, the following ointment will be found very useful:

℞. Acid Salicyl........................gr. x,
Ungt. Zinc Oxid....................
Ungt. Diachyli......................*aa* ℥ss.
*M.* Sig.

To this, there can be added often with benefit, carbonate of lead (grs. x–xx ad. ℥i), or ichthyol, 1 to 2 per cent. in strength. Should the surface be completely bereft of horny epidermis and profusely weeping, the case will often prove troublesome and rebellious. Such conditions are perhaps more often met with on the legs, and usually complicated with varicose veins. In their treatment, rest in bed and elevation of the leg are to be insisted upon, or when that is not practicable, thorough bandaging with gauze or muslin. I cannot say that I favor the rubber bandage, as I have seen it so often produce or render an eczema worse, and besides, I have always been able to obtain as good a result with the muslin ones. For the local treatment of these cases, I would especially recommend ichthyol with oxide of zinc ointment, and the strength of 2 to 5 per cent. The weeping born of the affection is also frequently met with associated with a want of keratofication, the surface being covered with shreddy, whitish masses of ephithelium, which can easily be rubbed off with the finger, and when this deficient hornification of the epithelial cells of the rete malpighii exists, the result obtained from the use of a 2 to 3 per cent. aqueous solution of ichthyol, or of one containing calamine and zinc oxide, will often be surprising.

The lotion must, however, be kept constantly in contact with the diseased surface, by means of lint or canton flannel soaked in it. In the troublesome intertrigo of infants and young children, I have found a 1 to 2 per cent. aqueous solution of ichthyol gives far and above the best results of any applications which can be made. So successful has it been, that it is used exclusively in my service in the New York Infant Asylum, on such cases. The affected surfaces must be kept continually moist by means of absorbent cotton soaked in the liquid.

Supposing, however, that the irritability of the eczematous patches, the acute attack or the profuse weeping have been removed, and the surface has become converted into a simple squamous eczema, what treatment should be instituted? We have now to deal with the products of the inflammation, the thickening and the infiltration, and for that purpose, tar and mercurial preparations occupy the first rank. The tars, however, are the most efficacious, acting almost as a specific in the majority of the cases of eczema, by promoting the absorption of the infiltration and by allaying the itching. Objectionable on account of their odor and color, they nevertheless retain their position, owing to their value, notwithstanding that many substitutes have been proposed for them. A certain amount of care must be taken in prescribing them, however, for individual idiosyncrasies will be met with. Only lately I have seen a most severe acute eczema produced by an ungt. containing ungt. picis liq., ʒi.; ungt. oxidi zinci, ʒvii., the eruption being limited strictly to surface upon which it was applied. When active inflammatory symptoms are present, tar should not be used, nor when weeping is present or there exists considerable absence of horny epidermis, nor without previous testing of the patient's skin over a small area should it be applied over the entire body, owing to its possible irritating effects and to the danger of absorption, carbolic acid poisoning etc. The tars, which can be recommended in eczema, are the oil of cade, the oleum rusci or white birch tar, and the pix liquida. The latter has proved, in my hands, to be certainly the most generally serviceable, not being so irritating as the oil o[illegible] cade, nor so disagreeable in odor and color as the oleum rusci[illegible] The pix liquida is usually used by me in the form of the officina[illegible] ungt. picis liquidæ, either full strength or diluted with some othe[illegible] ointment, according to the necessities of the case. If the ecze[illegible]matous surface is somewhat irritable, it can be combined wit[illegible] oxide of zinc ointment in the proportion of ungt. picis, ʒi. or ʒij. ungt. zinc oxid. q. s. ad. ℥i., and progressively increased with th[illegible] decrease in the inflammatory reaction of the affected skin[illegible] Rarely, however, have I ever found it necessary to use the ungt[illegible] picis liq. in greater strength than in equal parts with the zin[illegible] oxide. Should the thickening and infiltration of the skin be ver[illegible] marked, the ungt. picis, in combination with ungt. diachyli, wi[illegible] prove very useful, owing to the softening effect of the latter[illegible] The action of the salve can be, however, increased by the addi[illegible]tion of a few grains of acid salicylic (grs. v—x in ℥i).

Though for general use and efficacy the pix liquida has al[illegible]ways seemed to me superior to the other tars, still under certai[illegible] circumstances the ol. cadeni or rusci can be substituted for i[illegible]

with advantage. This is especially the case when there is a vei marked inactive condition of the skin, or a very great degree ( thickening, and nothing to be lost by the possible starting up ( a slight *acute exacerbation* of the eczema. In these cases tl ol. cadeni will often be serviceable, owing to its stimulatin properties. It can be applied either mixed with equal parts ( ol. oliv. or lini, or again in alcohol, or it can be used pure ( again in an ointment.

℞. Ol. Cadeni.................................ʒi-ʒiij.

Ungt. Zinc Oxid.......................... ℥i.

*M.* Sig.

Sometimes a more rebellious eczema is met with in female especially situated on the occiput at the junction of the hai scalp with the neck, a favorite place also for the eczema, resultin from pediculi capitis. But in the cases referred to, this cau can be readily excluded, owing to the absence of any nits or ( the lice themselves. The eruption has been seen by me usual to resist every form of treatment devised, and to yield only t the oil of cade, ʒii–ʒiij in ℥i of ointment, and I would cons( quently recommend its application in such cases. The oleu rusci is also very beneficial at times, but owing to its ver black color and strong, pungent odor, it can only be prescribe occasionally. I have found it particularly good in cases ( chronic papular eczema in adults when accompanied by consi( erable thickening and intense itching. It can be used either the form of an ointment or mixed with oil, ʒss to ʒi or more t the ℥i, but it seems to act better when combined with alcohc ʒss to ʒii to the ℥i. When the solution is painted over the al fected surface, the alcohol evaporates and the tar remains as paint which cannot be removed easily by ordinary rubbin When this solution is used, it is advisable to give the patient starch or alkaline bath once or twice a week. Chronic papul eczema is also met with quite often in children, and for the la few years I have treated them altogether with ichthyol locall not finding that the effect of tarry preparations was very goo( Usually beginning with grs. xv of ichthyol to ungt. diach ℥i, the strength has been rapidly increased to grs. 30, 40, 60 [illegible]

the ℥i, and the treatment has usually caused the objective and subjective symptoms to disappear rapidly. As an adjunct to the tar treatment, the use of soap is often of service, but it must be limited to those patches of the disease, which are very much thickened and inactive, the object being to light up an inflammatory reaction, to accelerate the change in nutrition and to hasten absorption of the inflammatory products. For this purpose sapo viridis is to be recommended, or in a more elegant form, the spts. saponis kalinus. The affected surface should be well washed with either one of these, and when dried the ointment in use is to be immediately applied. In chronic eczemas of the palms and soles, and of the fingers, this procedure will be found especially beneficial. Should, for some reason or other, tar not be well borne, or be strongly objected to, recourse may be had to mercurial preparations. Of these the white precipitate will be the most frequently of service, but the red oxide and the nitrate are also at times beneficial. The ammoniated mercury can be used in the strength of grs. x–l in the ungt. simp., or zinc oxid ℥i, and is especially good in scaly eczema of the scalp and face; the red oxide and nitrate when ordered should be applied with care, owing to their irritating action. The strength of each need not, as a rule, be more than grs. x–xxx to the ℥i. In addition to these B. naphthol gr. v–xl, ad. ℥i may be used, or chrysarobin collodium, 10 per cent. may at times be of value, but I have rarely found it necessary to use them. The chronic pustular or impetiginous eczema is one also seen frequently in children and young adults, and the fact that there is a secondary infection present must be borne in mind in these cases as well as when their eruption has occurred acutely. Such eczemas require, consequently, antiseptic treatment, and after the crusts have been removed by some of the means already mentioned, this may be obtained from a 5 to 10 per cent. ichthyol oil, or ungt., or boric acid 1-4, etc. A most serviceable application in these chronic pustular eczemas has been, however, a combination recommended years ago by Biett, which is approximately constituted as follows:

℞.—Ungt. Hydrarg. oxid. rub................ʒi–ʒii.

Ungt. sulphuris..........................ʒiii.

Ungt. oxid. zinc...........................*ad* ℥i.

*M.* Sig.

This form of eczema also occurs at times in a discrete form over the cutaneous surface. In such cases great benefit can be obtained by bichloride of mercury baths, 1-1000 or weaker, according to the age of the patient and the irritability of the skin. After such a bath a salicylic acid ointment is all that is necessary.

Allow me to add a few words in regard to the use of water in eczema. As a rule, plain water is injurious and almost invariably renders an attack worse. Especially is this the case in acute eczema and where there is much inflammation and exudation, and the fact is constantly observed that an eczema progressing favorably in every way under treatment, will relapse to its original condition after the injudicious use of water in some form or other. Patients should therefore always be warned against it, and its application should be incontinently stopped in every case. Still, under certain conditions and in certain stages, baths will be not only beneficial to the skin, but very grateful to the patient. They can be given once or twice a week. The cases in which this will be advisable are those of chronic inactive eczema, the papular and very pruritic ones, and also in the scaly conditions remaining after the subsidence of an acute attack. The baths may be either general or local, their temperature lukewarm, not hot, and they may be alkaline, or starch and alkaline, or starch alone. For an alkaline bath there should be added to 30 gallons of water

Sodii Bicarb..........................................℥xii.
Kali Bicarb...........................................℥vi.
Sodii Borat...........................................℥ij.
*M.* et S.

To this quantity one pound of starch may be added, should it be desired. My preferences, however, are for the starch baths alone or if it is necessary to remove from the skin much accumulation of grease and scales, then I add only the bicarbonate of soda.

The starch bath can easily be made by instructing the patient to add to the water such a quantity of raw starch as when dissolved will make the water milky in color. The best time for giving the bath is in the evening before retiring, and the patient may remain in it as long as 15 minutes. After being thoroughly dried he should be carefully rubbed all over with the ungt. used. Irritative baths are of doubtful value in eczema in any of its forms, and for that reason sulphur and mineral baths of all kinds had best be avoided, and the patient discouraged from taking them.

The treatment of eczema, which has been given, can only be regarded as a general outline of what is needed in the care of examples of this cutaneous disease. It is not claimed that by means of the remedies recommended all cases will be cured, or that there are no other forms of treatment which are applicable. On the contrary, many other procedures are, without question, fully as good, but my object having been to give, from my own experience only those which have proved the most generally serviceable and beneficial in the majority of cases, the others have been omitted. The use of the rubber base plasters, the gelatines, the particular and special remedies and treatment of seborrheic eczema, etc., could have also been added, but all these would require too much time and space, and consequently will have to be relegated to a more fitting opportunity. It happens to no one to cure every case of eczema, but the great majority can be caused to disappear and give place to a healthy skin. Such has been the result of my experience with the treatment delineated here.

*7 W. 31st St.*

